# A386G transition in *DAZL* gene is not associated with spermatogenic failure in Tamil Nadu, South India

**DOI:** 10.4103/0971-6866.42322

**Published:** 2008

**Authors:** J. Poongothai, T. S. Gopenath, S. Manonayaki

**Affiliations:** Research Associate, Center for Computational Engineering and Networking, Amrita Vishwa Vidyapeetham, Ettimadai, Coimbatore, India; 1 Human Biologicals Institute, Ooty (Division of Indian Immunologicals Ltd., Hyderabad, India), India; 2 Department of Animal Science and Biotechnology, Emerald Heights College for Women, Ooty, India

**Keywords:** *DAZL*, *DAZ*, microdeletions, normozoospermia, Tamil Nadu

## Abstract

The *DAZ*-like (*DAZL*) gene located on the short arm of autosomal chromosome 3 (3p24), an essential master gene for the premeiotic development of male and female germ cells, is the father of the Y-chromosome *DAZ* gene cluster and encodes for RNA-binding proteins. Reported instances of positive association of *DAZL* gene mutations with infertility in men have been found in a Taiwanese population but not in Caucasians. There is no study from Tamil Nadu, South India, to demonstrate the role of *DAZL* gene in male infertility; we, therefore, analyzed a total of 287 men, including 147 infertile and 140 normozoospermic fertile controls from rural areas of Tamil Nadu, South India, to assess the phenotypic effect of *DAZL* mutations in this region of the world. Interestingly, all our samples showed absence of the A386G (T54A) mutation that was found to be associated with spermatogenic failure in the Taiwanese population. Therefore, we suggest that the A386G (T54A) mutation is not associated with male infertility in Tamil Nadu, South India.

## Introduction

Infertility is a major health problem today, affecting ~10–15% of married couples. Male infertility accounts for ~50% of the cases, with quantitative or qualitative abnormalities of sperm production leading to spermatogenic failure. Genetic abnormalities, as well as numerical and structural chromosomal abnormalities, have been identified in men with unexplained oligozoospermia (1–20 × 10^6^spermatozoa / ml) and azoospermia (no sperms in the ejaculate).[1] However, recent evidence suggests that a variety of newly discovered genetic causes may be associated with idiopathic infertility.

The *DAZ* gene family is located in the distal euchromatic part of the long arm of the human Y chromosome (Yq11.23) in the so-called *AZFc* (AZoospermia Factor c) region. Among cases with Yq microdeletions, deletion involving the *DAZ* gene family is the most frequent finding. The *DAZ* gene is believed to have been transposed to the Y chromosome during primate evolution after the divergence of the New World and Old World monkeys, i.e. 35 × 10^6^ years ago,[2,3] from the ancestor gene DAZ-Like (*DAZL*) located on the short arm of autosomal chromosome 3 (3p24), arose ~30-40 million years ago. *DAZL* is highly homologous to the *DAZ* gene clustered on the Y chromosome, with 83% similarity in the coding region of the cDNA.[2–4] *DAZ* and *DAZL* are transcribed exclusively in the germ line and encode RNA-binding proteins of the highly conserved RNA-recognition motif (RRM) class. The *DAZL* gene has been demonstrated to be an essential master gene for the premeiotic development of male and female germ cells.[5]

Lilford *et al*,[6] suggested that up to 60% of undiagnosed male infertility are due to autosomal recessive mutations. So it is possible that a defective autosomal *DAZL* gene may be responsible for spermatogenic defects in some cases and that the genetic defect is inherited in an autosomal recessive fashion.[6] A mutation at nucleotide A386G in exon 3 of the *DAZL* gene in some infertile patients in a Taiwanese population was observed by Teng *et al*.[7] There are, however, no other reported instances of a positive association of *DAZL* gene mutations with infertility in men.

Whether *DAZL* plays a crucial role in spermatogenesis in humans merits investigation. There has been no study on infertile men from Tamil Nadu, South India, that has attempted to demonstrate the role of *DAZL* gene in male infertility. We, therefore, analyzed 147 infertile and 140 normozoospermic fertile controls from rural areas of Tamil Nadu, South India, to assess the phenotypic effect of *DAZL* mutations.

## Materials and Methods

### Patient selection

This study was conducted on subjects attending infertility clinics in Erode and Nilgiris Districts of Tamil Nadu, South India. We collected samples of blood from 45 infertile men, semen samples from 72 infertile men, and paired samples (blood and semen of the same patient) from 30 infertile men; 140 normozoospermic (> 20 million sperms / ml of semen) males (10 paired samples) of proven fertility served as controls. All the procedures followed were in accordance with the ethical standards of the Center for Cellular and Molecular Biology, Hyderabad.

Semen samples were obtained by masturbation on two different occasions, separated by a 3-week interval, following a 3-day period of sexual abstinence. Semen samples were allowed to liquefy for 30 min at 37°C. Sperms in the ejaculates were analyzed for their motility, number, and morphology as per the guidelines of the WHO, 1999.[8] Written informed consent was obtained from all participants in this study.

### DNA extraction and polymerase chain reaction

DNA was extracted from 10 ml of peripheral blood[9] and from semen[10] using standard procedures. Primers for exon 3 of *DAZL* were designed and synthesized (forward: 5′-TGAAAGAAATTAACACAGCAACAA-3′; reverse: 5′- GGGGGAGAAATTGTCACATCAT-3′). DNA samples were analyzed for the presence of the exon 3 of *DAZL* by polymerase chain reaction (PCR) in a 0.2-mL thin-wall tube under the following conditions: 50 ng of DNA, 1.2 mM MgCl_2_, 200 µM dNTPs, 5.0 pMol of each specific primer, and 1 IU AmpliTaq Gold in a 10 µL reaction volume. Amplification was carried out in a MJ Research Thermal Cycler (Waltham, MA, USA) using the following cycling conditions: after an initial denaturation step at 94°C for 5 min, cycle parameters were 94°C for 1 min, 60°C for 1 min, and 72°C for 60 sec for 30 cycles, with a final extension of 72°C for 10 min. Amplified products were quantified by 2% agarose gel electrophoresis.

### Sequencing DAZL from infertile males

PCR products were optimized for sequencing.^10^] Sequencing of PCR products was carried out using 100 ng (2.0 µL) of PCR product and 2 pmol (0.5 µL) of primer (forward and reverse, separately), 4 µL of BigDye Terminator Ready Reaction kit (Perkin Elmer), and 3 µL of double-distilled water to adjust the volume to 10 µL. Cycle sequencing was carried out in a GeneAmp 9600 thermal cycler (Perkin Elmer) employing the following conditions: 30 cycles at 96°C for 10 sec, at 50°C for 5 sec, and at 60°C for 4 min. Extended products were purified using the protocol described by Thangaraj *et al*.[11] Purified samples were dissolved in 10 µL of 50% Hi-Di formamide and analyzed in an ABI 3700 automated DNA analyzer (Perkin Elmer). The *DAZL* sequences of the infertile and normozoospermic fertile control men were edited and compared with the reference sequence using AutoAssembler^™^ software (Applied Biosystems, Foster City, CA, USA).

## Results

**Figure 1:**
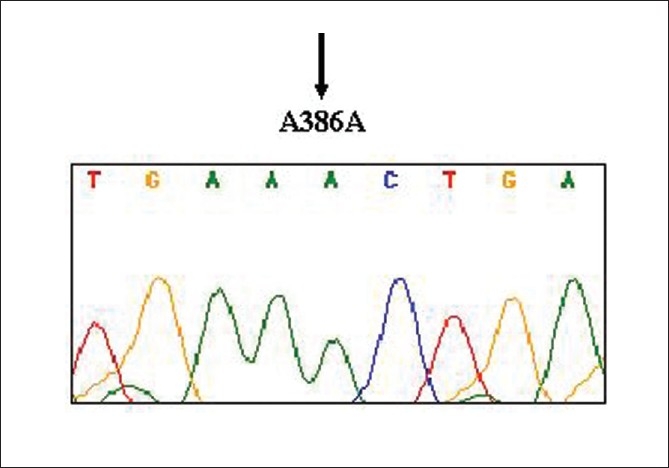
Electrophorogram of exon 3 of *DAZL* gene showing homozygous wild-type (A/A) allele

The seminogram of 147 infertile men ranged from azoospermia to oligozoospermia. There were 69 oilgozoospermic (1-20 × 10^6^ spermatozoa / ml), 50 asthenozoospermic (> 60% of nonmotile sperms), 24 oligoasthenozoospermic, and 1 each of varicocele (normal count), azoospermic (no sperms in the ejaculate), teratozoospermic (> 40% of abnormal sperms), and necrozoospermic (100% dead sperms) infertile men. Analysis of exon 3 of the *DAZL* gene in a total of 287 men, including 147 infertile and 140 normozoospermic control men of Tamil Nadu, South India, revealed the complete absence of the previously reported A386G mutation in the *DAZL* gene. Figure 1 depicts the electrophorogram of exon 3 of the *DAZL* gene showing homozygous wild-type (A/A) allele at position 386.

## Discussion

The large majority of microdeletions in infertile patients occur in the *AZFc* regions, where the *DAZ* gene cluster is located. In spite of this, there were few reports giving the evidence of the involvement of the autosomal gene *DAZL* in spermatogenesis. In many species, *DAZL* homologs are essential for the differentiation of germ cells. For example, the loss of *Boule* function results in meiotic arrest and azoospermia in drosophila. The spermatogenic defects of *Boule* flies could be partially rescued by the *Xenopus Xdazl* gene.[12] Similarly, sterility of Dazl knockout mice was also partially rescued by the human *DAZ* gene. These findings suggest a high degree of functional conservation between the *DAZ* and *DAZL* genes. To date there has been no consensus in the literature regarding the role of autosomal *DAZL* gene in human male infertility.

In humans some very limited studies have been carried out on the *DAZL* gene and its role in male infertility. Teng *et al*,[7] identified two polymorphic sites in the *DAZL* gene in a Taiwanese population. One was A260G (T12A) of exon 2, which was seen in 3.5% and 2.59% of infertile and fertile men, respectively. Another variant was A386G, with a change of threonine to alanine (T54A) in exon 3, which was seen in 7.39% of the infertile and 0.86% of the fertile men. Thangaraj *et al*,[13] reported two polymorphic sites in the *DAZL* gene in an Indian population comprising people of different ethnic / linguistic origins: A260G (T12A) of exon 2 in 8.1% and 7.4% of the infertile and fertile control men, respectively, and A437G polymorphism in four fertile and one infertile man. Another variant, A386G, leading to a change of threonine to alanine (T54A) in the exon 3, was not found in infertile men or fertile controls in the Indian subcontinent. Similarly, our study on 147 infertile men and 140 normozoospermic fertile control men from rural areas of Tamil Nadu, South India, belonging to Dravidian linguistic family, for exon 3 showed complete absence of A386G (T54A) mutation in both infertile and fertile normozoospermic control men.

Three independent studies conducted recently also did not find the T54A mutation in Caucasian populations.[14–16] Similar results were observed in a Japanese population by Yang *et al*.[17] The distribution of the T54A polymorphism may be restricted to a narrow area, including Taiwan, as reported by Teng *et al*.[7] As the T54A mutation was observed only in the Taiwanese population and was absent in Caucasian populations, Bartoloni *et al.*[14] suggested that this mutation may play a role in infertility only in the Taiwanese population. Thangaraj *et al.*[13] suggested that these mutations in the *DAZL* gene may not be associated with male infertility in the Indian subcontinent. Our study on infertile and fertile normozoospermic men of Tamil Nadu, South India, revealed complete absence of the T54A mutation in exon 3 of *DAZL*. Therefore we suggest that the A386G (T54A) mutation is not associated with male infertility in Tamil Nadu, South India.
